# Supplementary material for: Content-rich biological network constructed by mining PubMed abstracts
Source: BMC Bioinformatics. 2004 Oct 8;5:147. doi: 10.1186/1471-2105-5-147 (PMC528731; doi:10.1186/1471-2105-5-147)
Supplement: Additional File 2 — The original results of the above study (non-essential files are deleted to keep the file size under the limit set by BMC bioinformatics). [file 1471-2105-5-147-S2.bz2 › chilibotAdditionalFile2/dip40/567ID11121055E10931/html/FGF10_FGFR2.html]

 


 **FGF10** and **FGFR2** 
  
Found 47 abstracts in PubMed, retrieved 40.  
 

 What does Google say? 
 PDF only 
| .edu only 

---

**Interactive relationship** (e.g. stimulation, inhibition, etc)

**Neutral relationship**- FGF7 and  **FGF10**  activate only the b isoform of  **FGFR2**  FGFR2b.  Ref: 12591959 Proc Natl Acad Sci U S A, 2003
- Interactions between  **FGF10**  and the IIIb isoform of FGFR 2  [ **FGFR2** ]  appear to be crucial for the induction and growth of several organs, particularly those that involve budding morphogenesis.  Ref: 10934262 J Neurosci, 2000
- **FGF10**  acts as a major ligand for FGF receptor  [ **FGFR2** ]  2 IIIb in mouse multi organ development.  Ref: 11062007 Biochem Biophys Res Commun, 2000
- FGF 10  [ **FGF10** ]  actions are dependent on their binding to the iiib form of FGF receptor  [ **FGFR2** ]  2  **FGFR2**  iiib, also known as keratinocyte growth factor receptor  [ **FGFR2** ]  KGFR  [ **FGFR2** ] .  Ref: 11404772 J Nippon Med Sch, 2001
- It is likely, therefore, that whereas during early development  **Fgfr2**  interacts with Fgf4, in limb and lung development interactions between  **Fgf10**  and  **Fgfr2**  may be required.  Ref: 10518547 Proc Natl Acad Sci U S A, 1999
- FGF 10  [ **FGF10** ]  is a secreted growth factor that binds to FGF receptor  [ **FGFR2** ]  2 IIIb, which is expressed in prostatic epithelial cells and thus can potentially act as a growth factor for these cells.  Ref: 10951499 Prostate, 2000
- These findings suggest that FGF 7 and FGF 10  [ **FGF10** ]  contribute to the regeneration and differentiation of acinar cells and angiogenesis in AP through KGFR  [ **FGFR2** ] .  Ref: 12565793 Exp Mol Pathol, 2002
- We show that the S252W mutation allows the mesenchymal splice form of  **FGFR2**  FGFR2c to bind and to be activated by the mesenchymally expressed ligands FGF7 or  **FGF10**  and the epithelial splice form of  **FGFR2**  FGFR2b to be activated by FGF2, FGF6, and FGF9.  Ref: 11121055 Proc Natl Acad Sci U S A, 2000
- Both fibroblast growth factor 10  [ **FGF10** ]  and keratinocyte growth factor bind with high affinity to the tyrosine kinase keratinocyte growth factor receptor  [ **FGFR2** ] .  Ref: 11286634 J Invest Dermatol, 2001
- Immunoprecipitation western blot analysis revealed that fibroblast growth factor 10  [ **FGF10** ] , similarly to keratinocyte growth factor, is able to induce tyrosine phosphorylation of keratinocyte growth factor receptor  [ **FGFR2** ]  and of cellular substrates such as PLCgamma.  Ref: 11286634 J Invest Dermatol, 2001
- FGF1, FGF7, FGF9,  **FGF10** , FGF11, and FGF18 were also expressed in the pancreas at varying time points and levels, as well as FGF receptor  [ **FGFR2** ]  forms FGFR1b, FGFR1c, FGFR2b, FGFR2c, FGFR3b, and FGFR4.  Ref: 12666204 Dev Dyn, 2003
- Keratinocyte growth factor receptor  [ **FGFR2** ]  KGFR  [ **FGFR2** ] , also known as fibroblast growth factor receptor FGFR 2  [ **FGFR2** ]  IIIb, is located in many types of epithelial cells and is activated by four known ligands FGF 1, FGF 3, FGF 7 also known as KGF and FGF 10  [ **FGF10** ]  that are predominantly synthesized by mesenchymal cells.  Ref: 12608893 Pathol Int, 2003

**Non-interactive relationship** (e.g. studied together, co-existance, homology, etc.)

- **FGF10**  and FGFR 2  [ **FGFR2** ]  IIIb mRNAs showed distinct, largely nonoverlapping expression patterns in the undifferentiated otic epithelium.  Ref: 10934262 J Neurosci, 2000
- However, treatment with exogenous  **FGF10**  or antisense  **Fgf10**  did not change Shh and  **FgfR2**  mRNA levels in the lungs.  Ref: 11287183 Mech Dev, 2001
- **FGFR2**  iiib mRNAs were expressed in these cells and the recombinant FGF 10  [ **FGF10** ]  1 ng ml increased the growth rate of COLO 205 cells.  Ref: 11598623 J Nippon Med Sch, 2001
- Differences in the level and pattern of expression of mRNA for KGF, FGF 10  [ **FGF10** ] , and the KGFR  [ **FGFR2** ]  during postnatal development of the mouse mammary gland are a result of morphological development, changes in the ratio of stroma to epithelium, and hormonal regulation of cell differentiation.  Ref: 10911393 Mol Reprod Dev, 2000
- Fibroblast growth factor receptor 2  [ **FGFR2** ]  IIIb acts upstream of Shh and Fgf4 and is required for limb bud maintenance but not for the induction of Fgf8,  **Fgf10** , Msx1, or Bmp4.  Ref: 11180951 Dev Biol, 2001
- These findings indicate that FGF 10  [ **FGF10** ]  and its receptor,  **FGFR2**  iiib expression in colorectal adenocarcinoma cells and FGF 10  [ **FGF10** ]  may contribute to the growth of cells of this type.  Ref: 11598623 J Nippon Med Sch, 2001
- Both CaSki and ME 180 cells expressed  **FGFR2**  iiib mRNA, while only CaSki cells expressed FGF 10  [ **FGF10** ]  mRNA and protein.  Ref: 11404772 J Nippon Med Sch, 2001
- In the present study, the expression of FGF 10  [ **FGF10** ]  and  **FGFR2**  iiib in five cultured human colorectal adenocarcinoma cell lines COLO 205, DLD 1, HCT 15, SW 480 and WiDr and the localization of FGF 10  [ **FGF10** ]  messenger RNA mRNA and its protein in human colorectal cancer tissues from 10 patients were determined.  Ref: 11598623 J Nippon Med Sch, 2001
- Our results, in comparison with functional studies in other systems, suggest that FGF 2 FGFR 1, FGF 8 FGFR 2  [ **FGFR2** ]  IIIc and FGF 10  [ **FGF10** ]  FGFR 2  [ **FGFR2** ]  IIIb signaling have different paracrine and juxtacrine functions during SMG initial bud formation and branching.  Ref: 11731698 Cells Tissues Organs, 2002
- In the present study, the expression of FGF 10  [ **FGF10** ]  and  **FGFR2**  iiib messenger RNA mRNA in two different human uterine cervical cancer cell lines CaSki and ME 180 were examined.  Ref: 11404772 J Nippon Med Sch, 2001
- Recently it was shown that  **Fgf10** , a major ligand for the IIIb isoform of fibroblast growth factor receptor 2  [ **FGFR2** ]  Fgfr2b, has an important regulatory role in early pancreas development.  Ref: 12559489 Mech Dev, 2003
- **FGF10**  and FGF receptor  [ **FGFR2** ]  FGFR 2  [ **FGFR2** ]  immunostaining showed a similar epithelial expression pattern.  Ref: 12697038 J Endocrinol, 2003
- Its absence did not prevent expression of Fgf8,  **Fgf10** , Bmp4, and Msx1, but did prevent induction of Shh and Fgf4, indicating that they are downstream targets of  **FgfR2**  IIIb activation.  Ref: 11180951 Dev Biol, 2001
- Semiquantitative reverse transcription polymerase chain reaction RT PCR and in situ hybridization analyses were used to study temporal and spatial alterations in expression of mRNAs for growth factors FGF 7, FGF 10  [ **FGF10** ] , HGF, IGF I, IGF II and their respective receptors FGF receptor  [ **FGFR2** ]  or FGFR2IIIb, c met, and IGF IR in the developing neonatal ovine uterus.  Ref: 11259272 Biol Reprod, 2001
- Highly similar lung and limb phenotypes were detected recently in the loss of function mutation of  **Fgf10** , a ligand of  **Fgfr2** .  Ref: 10518547 Proc Natl Acad Sci U S A, 1999
- Fgf7 and  **Fgf10** , the main ligands for  **FgfR2**  IIIb, are expressed in the mesenchyme surrounding the thymic epithelial primordium, and  **Fgf10**  deficient mice also exhibit impaired thymic growth.  Ref: 11489975 J Immunol, 2001
- In early limb buds, down regulation of Fgf receptor  [ **FGFR2** ]  1 and  **Fgf10**  expression in the mesenchyme occurs concurrent with loss of Fgf4 and Fgf8 expression in the AER.  Ref: 12175489 Mech Dev, 2002
- In later development, FGF3 or  **FGF10**  acting on FGF receptor  [ **FGFR2** ]  2b is likely to be involved in development of the walls of the cochlear spaces, while FGF receptor  [ **FGFR2** ]  3 is involved in differentiation of the pillar cells of the organ of Corti.  Ref: 11914524 Audiol Neurootol< MedlineTA>Audiol Neurootol, 2002
- The expression of the KGF receptor KGFR  [ **FGFR2** ]  and its stromal ligands, KGF and FGF 10  [ **FGF10** ] , was compared during mouse mammary gland development.  Ref: 10911393 Mol Reprod Dev, 2000
- In the Twist forelimb bud,  **Fgf10**  expression is reduced, Fgf4 is not expressed, and the domain of Fgf8 and  **Fgfr2**  expression is altered.  Ref: 12142027 Dev Biol, 2002
- In contrast, FGF receptor  [ **FGFR2** ]  2IIIb, the high affinity receptor for both FGF 10  [ **FGF10** ]  and FGF 7, was expressed exclusively in luminal epithelium, glandular epithelium, and placental trophectoderm.  Ref: 10952944 Biol Reprod, 2000
